# Work Hard, Party Harder: Drug Use and Sexual Behaviour in Young British Casual Workers in Ibiza, Spain

**DOI:** 10.3390/ijerph111010051

**Published:** 2014-09-26

**Authors:** Danielle Kelly, Karen Hughes, Mark A. Bellis

**Affiliations:** Centre for Public Health, Liverpool John Moores University, Henry Cotton Building, 15-21 Webster Street, Liverpool, L3 2EJ, UK; E-Mails: k.e.hughes@ljmu.ac.uk (K.H.); m.a.bellis@ljmu.ac.uk (M.A.B.)

**Keywords:** drug use, alcohol, sexual behaviour, sexual health, casual workers

## Abstract

*Background*: Every summer, young people flock to nightlife-focused holiday resorts around the world to find casual work. Despite being exposed to hedonistic environments, often for several months, little is known about their substance use, sexual activity and health service needs over this extended amount of time abroad. *Methods*: A short anonymous questionnaire examining alcohol and drug use, sexual behaviour and use of health services was administered to young British casual workers aged 16–35 in San Antonio, Ibiza (n = 171). *Results*: 97.7% of casual workers used alcohol in Ibiza, and the majority (85.3%) used drugs. Almost half (43.5%) of all participants used a drug in Ibiza that they had never used in the UK. Most casual workers arrived in Ibiza without a partner or spouse (86.5%). Of these, 86.9% had sex during their stay and 50.0% had unprotected sex; often while under the influence of alcohol. Only 14.3% of those having unprotected sex with a new partner sought a sexual health check-up in Ibiza, although 84.1% intended to do this on their return to the UK. *Conclusion*: Substance use and sexual risk taking is widespread among young British casual workers in Ibiza. Such international nightlife resorts represent key settings for substance-related health and social problems, and for the international spread of sexually transmitted infections. Addressing the health needs of casual workers and the environments that permit and promote their excessive behaviour requires collaboration between authorities in home and destination countries and the tourism industry.

## 1. Introduction

Young people who holiday in nightlife-focused resorts often increase their engagement in health risk behaviours during their stay, including alcohol use, drunkenness, drug use and risky sexual behaviour [1,2,3]. Holiday periods represent a time of excess and experimentation for young people, when they are free from usual social restraints and responsibilities and have increased opportunity to meet new people and try new things. Visiting a nightlife-focused holiday resort can submerge individuals in environments where hedonistic partying is the norm; alcohol is typically heavily promoted, drugs can be widely available and entertainment is often highly sexualised [4]. Sexual activity can increase due to increased opportunities to meet new sexual partners and the suspension of normal social codes, leading to accelerated sexual relationships [5,6,7]. In a study of young British holidaymakers visiting Majorca and Ibiza in Spain, for example, over a quarter of those travelling without a sexual partner had had sex during their stay and a third of these individuals had unprotected sex. Drunkenness was widespread in both locations but more common in Majorca, where 95% reported having been drunk at least once during their holiday and over 60% on five or more days per week. However, drug use was strongly associated with Ibiza, where over half of young holidaymakers reported having used at least one illicit drug during their stay [8].

In comparison to studies on tourists, relatively little research has focused on the substance use and sexual behaviour of young people who extend their stays in holiday resorts through casual work. Studies have shown that casual workers often remain in these hedonistic environments for several months working for bars, nightclubs or other tourism-related industries in international resorts such as Spain [9], Bulgaria [10], Australia [11] and the Caribbean [12]. Casual workers have been identified as instrumental mediators in both creating a social arena of risk and influencing the behaviours of tourists in nightlife resorts, promoting excessive alcohol and drug use and sexualised activities [4,10,13]. A study comparing British casual workers in Ibiza with tourists found that casual workers were more likely to be drug users; with a greater proportion having used drugs both during their stay in Ibiza and in the UK in the 12 months prior to visiting the island. Although drug users who were casual workers used drugs less frequently than holidaymakers (likely due to the requirements of working), ecstasy at least, however, was consumed in greater quantities [9]. Increased levels of sexual risk taking were also found in casual workers, including unprotected sex and sex with multiple partners [13]. Casual workers therefore represent a high risk population that, due to their extended stay in a foreign country, is often beyond the reach of health services. Thus, here we examine substance use and sexual behaviour among young British casual workers in Ibiza, exploring use of new drugs in Ibiza, the involvement of alcohol in risky sexual behaviours and the use of health and sexual health services by casual workers.

## 2. Methods

A short questionnaire was developed based on a tool used previously in research [9,13]. The questionnaire measured casual workers’ basic demographics, reasons for choosing to visit Ibiza, if they had previously worked on the island, length of current stay, type and hours of work, and frequency of substance use (alcohol, tobacco, cannabis, ecstasy, amphetamines, ketamine and gammahydroxybutyrate (GHB) in Ibiza and in the last 12 months spent in the UK. Sexual activity in Ibiza was measured through questions asking the number and type of sexual partners casual workers had whilst in Ibiza; their use of contraception with these partners; and whether participants had unprotected or regretted sex under the influence of alcohol or drugs. Participants were also asked how many sexual partners they had in the last 12 months in the UK.

The target sample was young British people (aged 16–35) working in San Antonio, Ibiza, in July/August 2009. Casual workers were defined as those working in bars, nightclubs, restaurants, hotels, as holiday reps, ticket sellers, or in any other tourism-related environment. Participants were approached opportunistically in areas frequented by casual workers, such as beaches and bars during the day and early evening (before 9 pm). Potential participants were asked if they had time to fill in a short questionnaire (n = 221); those who had time (n = 199) were informed about the nature of the study and that it was anonymous and confidential. Consent was obtained orally from those who were willing to take part (n = 182). Participants were handed a questionnaire on a clipboard, a pen and an envelope and asked to self-complete the questionnaire while the researcher stood a small distance away to maintain confidentiality, allowing the participant to ask questions if required. The participant was then told to place the completed questionnaire into a sealed envelope and return this to the researcher. All sealed envelopes were returned to the UK at the end of the study period for analysis. Participants were excluded if they had been in Ibiza for longer than six months (n = 8), to ensure that the sample represented only seasonal workers and not individuals who were living in Ibiza for longer periods of time. For the purpose of this analysis, three individuals that provided no information on substance use or sexual behaviour were also excluded, leaving a final sample of 171 casual workers. Data were analysed using SPSS. Analysis used chi-squared and ANOVA with logistic regression to identify factors independently associated with sexual behaviour in Ibiza.

## 3. Results

Half of participants (52.6%) were female and mean age was 22.1 years. Mean length of stay in Ibiza at the point of survey was 10.3 weeks. Half (50.9%) of participants had worked in Ibiza previously. The most common occupations of casual workers in San Antonio were working in bars and nightclubs as waitresses or bar staff (30.4%), PR work promoting a bar or nightclub (24.0%) and selling tickets for nightclubs (18.1%), with a small percentage working in hotels or as holiday ‘reps’. The most common reasons for choosing to work in Ibiza were for its nightlife (73.7%), music (73.1%) and weather (71.3%; multiple options could be selected). Males were more likely than females to have chosen Ibiza for its music (84.0% *vs*. 63.3%, *p* = 0.002) drugs (61.7% *vs*. 20.0%, *p* < 0.001) and sex (51.9% *vs*. 15.6%, *p* < 0.001). There were no gender differences in age, length of stay, total intended stay or previous seasons worked.

**Figure 1:**
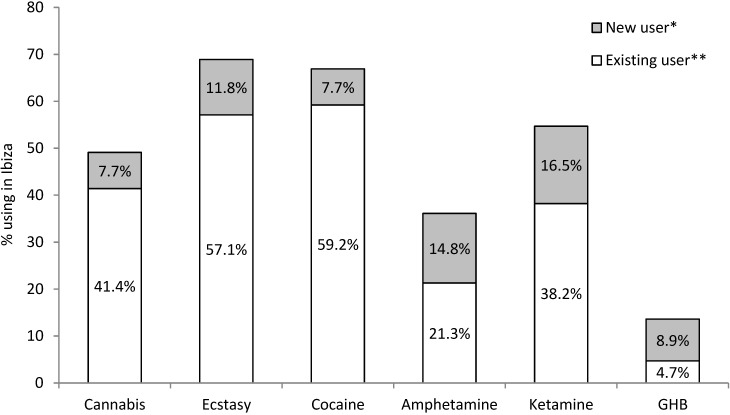
Prevalence of illicit drug use by casual workers in Ibiza, by new and existing drug users.

Note: * Never used the drug in the UK; ** Used the drug at least once in the UK.

Most (97.7%) participants had consumed alcohol in Ibiza and 96.3% of drinkers reported getting drunk at least once a week. Half (54.1%) reported smoking tobacco and 85.3% using illicit drugs. There were no gender or age differences in substance use. The most common drugs used were ecstasy (68.8% of participants) and cocaine (66.9%), followed by ketamine (54.7%), cannabis (49.1%), amphetamines (36.1%) and GHB (13.6%). Most (87.6%) of those that used drugs in Ibiza used more than one type of drug, and many (50.7% of drug users, 43.5% of all participants) reported using drugs in Ibiza that they had never used in the UK (Figure 1). Across all participants, 16.5% used ketamine in Ibiza having never used in the UK, and for amphetamine and ecstasy these figures were 14.8% and 11.8%, respectively. For GHB, 15 of the 23 participants who had used in Ibiza had never used the drug in the UK. Amongst those reporting use of specific drugs in both UK and Ibiza, frequency of use increased in Ibiza. Thus, whilst 98.8% of ecstasy users stated using the drug ≤once a week in the UK, in Ibiza this fell to 59.5%, with 10.7% using the drug 5+ days a week (Table 1).

The majority (86.5%, n = 147) of participants had travelled to Ibiza without a sexual partner (Table 2). Of those arriving with a sexual partner, 87.0% reported either one or no sexual partner in Ibiza. Thus, analysis of sexual behaviour was limited to the majority of participants arriving without a sexual partner. Of these single participants, 86.9% reported having sex in Ibiza and 71.7% reported more than one sexual partner. One hundred and twenty-six sexually-active single individuals provided further data on sexual activity. Half (50.0%) reported unprotected sex (without a condom) and a quarter (23.8%) reported unprotected sex with more than one partner. A third (37.3%) had sex that they later regretted. Over 90% of participants reporting either unprotected or regretted sex stated that they had been under the influence of alcohol when this occurred. Overall, participants’ sexual partners were most commonly other workers or tourists (reported by 71.4% and 69.8% of single sexually active participants, respectively). Almost a third (30.4%) had sex with someone not from their country of residence and almost a fifth (18.3%) with a local resident. Of the participants that arrived in Ibiza without a sexual partner or spouse and had sex (n = 126), 9.5% (n = 6) had same-sex sexual activity during their stay. Of those 9.0% (n = 6) were female, and 10.2% (n = 6) were male. Further analyses with these data were not conducted due to the small sample sizes but are listed here for completeness.

**Table 1:** Frequency of substance use in UK and Ibiza among those using in both locations.

Substances Used	Alcohol	Tobacco	Cannabis	Ecstasy	Cocaine	Amphetamine	Ketamine
*n*	*165*	*81*	*62*	*84*	*87*	*32*	*50*
**Frequency of use UK %**							
Once a week or less	42.4	19.8	70.5	98.8	93.1	100.0	98.0
2–4 days a week	47.3	11.1	18.0	1.2	5.7	0.0	2.0
5 or more days a week	10.3	69.1	11.5	0.0	1.1	0.0	0.0
**Frequency of use Ibiza %**							
Once a week or less	7.3	7.4	59.0	59.5	57.5	62.6	44.0
2–4 days a week	22.4	8.6	23.0	29.8	31.0	25.0	40.0
5 or more days a week	70.3	84.0	18.0	10.7	11.5	12.5	16.0
P between locations	<0.001	<0.001	<0.001	<0.001	<0.001	<0.001	<0.001

Note: GHB was not analysed due to low prevalence of use in UK.

In chi squared analysis, there were no associations between having sex, multiple sexual partners or unprotected sex and gender, age or employment characteristics. Longer length of stay was associated with having had sex and multiple partners (Table 2). Identifying music, drugs or sex as a reason for choosing to work in Ibiza was associated with having sex, whilst identifying sex as a reason was associated with multiple partners. Tobacco, cocaine and amphetamine use were associated with having sex, and amphetamine use with multiple partners. Illicit drug users were more likely to report sex and multiple sexual partners than non-users. Frequent drunkenness was strongly associated with both sex and multiple sexual partners. None of the factors examined had significant relationships with unprotected sex.

Factors with significant relationships in chi squared analysis were entered into logistic regression models. Here, odds of having sex were increased in those who: had been on the island for >10 weeks; identified sex as a reason for choosing to work in Ibiza; used amphetamines; and got drunk frequently. Odds of having multiple sexual partners were increased in those who: had been on the island >10 weeks; used tobacco; and got drunk frequently (Table 2).

**Table 2:** Factors associated with having sex and having multiple sexual partners among those arriving in Ibiza without a sexual partner *.

Variables	Variable Categories			Had Sex in Ibiza					
				%Yes ^a^	X²	*p*	AOR ^b^	95%CIs	*p*
Time spent in Ibiza by point of interview	Up to 10 weeks			80.0			Ref		
	More than 10 weeks			95.4	7.455	0.006	9.62	2.10–43.98	0.004
Reasons for choosing to work in Ibiza	Sex	No		82.1	5.554	0.018	Ref		
		Yes		96.0			8.27	1.39–49.08	0.020
	Drugs	No		81.4	5.617	0.018			
		Yes		94.9					ns
	Music	No		74.4	7.365	0.007			
		Yes		91.5					ns
Substance use in Ibiza	Amphetamine	No		81.6	5.182	0.023	Ref		
		Yes		94.7			5.47	1.18–25.31	0.030
Number of illicit drugs used	None			66.7					
	1–3			88.3					
	4–6			90.6	7.221	0.027			ns
Frequency of drunkenness in Ibiza	≤once a week			66.7			Ref		0.008
	2–4 times a week			84.0			5.92	1.33–26.41	0.020
	5+ times a week			94.6	11.767	0.003	11.37	2.35–55.01	0.002
				**Had Multiple Sexual Partners in Ibiza**					
				**%Yes**	**X²**	***p***	**AOR**	**95%CIs**	***p***
Time spent in Ibiza by point of interview	Up to ten weeks			65.0			Ref		
	More than 10 weeks			80.0	3.979	0.046	2.56	1.05–6.26	0.039
Substance use in Ibiza	Tobacco		No	60.6			Ref		
			Yes	82.1	8.195	0.004	3.19	1.35–7.53	0.008
	Cocaine		No	60.0					
			Yes	77.0	4.422	0.035			ns
	Amphetamine		No	65.5					
			Yes	80.7	3.899	0.048			ns
Number of illicit drugs used	None			44.4					
	1–3			73.3					
	4–6			79.7	8.792	0.012			ns
Frequency of drunkenness in Ibiza	≤once a week			38.1			Ref		
	2–4 times a week			66.0			3.05	0.95–9.82	0.062
	5+ times a week			85.1	19.081	<0.001	11.62	3.41–39.65	<0.001

^a^ Chi squared analysis. Only factors with significant associations shown; ^b^ Logistic regression analysis including all factors shown; AOR = adjusted odds ratio; Ref = reference category.

Only 34.1% of participants reported having received any health information on drugs, alcohol or sexual health in Ibiza. Under a third (28.9%) had visited a doctor or hospital during their stay. Of individuals arriving without a sexual partner and having unprotected sex on the island, only 14.3% reported having had a sexual health check-up in Ibiza. However, 84.1% reported that they would be seeking such a check-up on return to the UK.

## 4. Conclusion

Young casual workers in nightlife-focused resorts are a key at risk group for health and social harm. Among British casual workers in Ibiza, regular drunkenness was ubiquitous and 85.3% reported having used at least one illicit drug during their stay. Most casual workers used more than one drug type and around half had used a drug in Ibiza that they had never used in the UK. Given that casual workers were intending on staying an average 8.5 weeks longer on the island post-survey, this figure likely underestimates the actual prevalence of drug experimentation. Recruitment and relapse into drug use is a particular concern as individuals are likely to continue such levels of experimental drug use on return to the UK [2]. Most casual workers arrived in Ibiza without a partner or spouse and over two thirds of these reported having had more than one sexual partner by the time of interview. Half reported unprotected sex, which often occurred under the influence of alcohol. Despite such high levels of substance use and risky sexual behaviour, however, only 34.1% reported having received any relevant health information in Ibiza.

Scholars have previously commented that the behaviour of young tourists on holiday is simply an extension of leisure behaviours that take place at home every weekend [14,15], and this may be true of some holiday resorts. Nevertheless, the extended stay of casual workers in a heavily drug- influenced environment in Ibiza can act as a catalyst to the development of behaviours out of the ordinary, beyond those undertaken at home [4]. Ibiza’s reputation as a major international dance music destination likely attracts high-risk young people already engaged in, or interested in, recreational drug use. In our study, 53.5% of casual workers reported use of ecstasy and 55.3% cocaine in the UK prior to visiting Ibiza; far higher than the past year prevalence of these substances reported among young people in the general UK population (in 2009/10, ecstasy 4.3%, cocaine 5.5%; Home Office, 2010) [16]. However, our findings suggest that the culture into which casual workers are immersed in Ibiza fosters tendencies towards risk behaviour. Many casual workers, particularly those working in nightlife-related industries, receive free entrance to nightclubs, discounted drinks and socialise in bars, nightclubs and staff accommodation blocks after work. With many casual workers returning to Ibiza year after year, new arrivals are likely to be rapidly integrated into a scene led by other high risk workers who use drugs and know how to find them. The wide availability and accessibility of drugs in Ibiza is emphasised by the high prevalence of use and experimentation identified here. Association with other high-risk casual workers, tourists and bar owners may invoke peer pressure to use drugs, while long working hours and routine late night partying could mean substance use becomes an essential part of maintaining the pace. The consequences of such progression would include declining physical and mental health, while in some cases low wages and high social spend may lead to the supplementation of income through illicit means, including drug selling [4,17]. Despite this, there appears to be few relevant services available to advise, support or protect this high risk transient population, and few interventions to moderate their behaviour by authorities in either Ibiza or the UK.

In addition to the harms associated with drunkenness and involvement in drug cultures, our findings build on previous work identifying casual workers as being at high risk of sexual health problems [13]. Thus, we found that frequent drunkenness was a strong predictor of having multiple sexual partners and that sexual risk taking often occurred under the influence of alcohol. Alcohol use can facilitate sexual interactions by lowering inhibitions and raising confidence; yet at the same time reducing people’s ability to make informed decisions about having sex and using contraception [18,19,20]. Thus, of those arriving without a sexual partner, 93.4% of those reporting unprotected sex and 97.8% of those reporting sex they later regretted said this had occurred whilst they were under the influence of alcohol. Increased involvement in frequent casual sexual relationships whilst abroad could potentially affect the behaviours of young people on return to the UK, as risky sexual activity becomes normalised. Whilst most sexual partners identified in our research were casual workers and tourists, research elsewhere has identified that that young tourists are engaging in sex with prostitutes and other sex workers [3,21]. For that reason, future research should explore whether casual workers are engaging in paid sex markets during their stay. Despite widespread sexual risk taking, however, few casual workers that had unprotected sex with a new partner had received a sexual health check-up in Ibiza; although the majority stated that they would seek such a check-up on return to the UK.

There may be numerous reasons for the failure of young people to access health services following unprotected sex, including a lack of available services to access; a lack of knowledge of, or trust in, foreign healthcare systems; a reluctance among young people to interrupt their fun with knowledge of a sexually transmitted infection (STI); or even nonchalance about the impact of contracting STIs. Chlamydia, the most common STI affecting young people in the UK [22], is easily treatable with antibiotics and can be viewed by young people as trivial [23]. Poor awareness of the differences between chlamydia and more serious conditions such as HIV [24] may mean this blasé attitude extends to STIs in general. However, the delay in obtaining a sexual health check up in a highly sexualised environment creates opportunity for the rapid spread of any STI, and with sexual mixing involving various nationalities and local residents, for their international dissemination. With infections such as syphilis and gonorrhoea increasing in Europe [25], and in particular the emergence of drug-resistant gonorrhoea [26], casual workers in international nightlife resorts should be viewed as a critical target population for sexual health interventions.

Our findings suggest a lack of health information available for young casual workers in Ibiza. Only around a third of participants had received information on sex, alcohol or drugs whilst in Ibiza, despite their length of stay by survey averaging over 10 weeks. With high levels of substance use, experimentation with new drugs and widespread sexual activity among casual workers, the availability of information on health risks, harm reduction and where to go for health advice and treatment should be considered essential. Development and dissemination of such information should be co-ordinated through health and tourism authorities in both the host country and that of young people’s country of residence. For example, as well as disseminating health-related materials in resorts themselves, advice and information could be targeted to young people through relevant websites and other media catering for those planning to work in an international nightlife resort. Broader strategies could include the development of staff training programmes for individuals working in holiday resorts, covering issues such as health, safety (both personal and within the workplace), legal issues, and availability of health and other services. Casual workers are key figures in promoting and maintaining risk environments and influencing the behaviours of tourists. If casual workers become more responsible for their individual health and wellbeing, for example drinking less alcohol, this could potentially reduce excessive behaviours amongst their peers.

Like all research into sensitive subjects, our study may have been affected by under- or over-reporting of substance use and sexual behaviour. However, to limit this effect participants were assured of their anonymity and provided with a method of completing the questionnaire in private and returning it to the researcher in an unmarked sealed envelope. Use of different illicit drugs was self-assessed and with widespread drug experimentation in Ibiza, it is possible that some participants may have misidentified substances. Equally, recall issues may have affected reports of substance use frequency in the UK in the 12 months prior to arriving in Ibiza. Finally, sampling was conducted on a convenience basis and focused only on British casual workers in the resort of San Antonio, thus findings cannot be generalised to all British casual workers in this resort. However, the study does give an important snap-shot of the behaviour of casual workers in one of the most popular nightlife resorts in the world. Findings highlight the need for greater focus on both the health of this high-risk population and the environmental factors that amplify their health harming behaviours.
